# Deciding on thresholds for clinical relevance of PROMIS-upper extremity v2.0 in patients with upper extremity injury

**DOI:** 10.1007/s11136-026-04367-w

**Published:** 2026-08-29

**Authors:** Suus G. J. van Bruggen, Lore E. de Vreeze, Charlotte M. Lameijer, Frank W. Bloemers, Caroline B. Terwee

**Affiliations:** 1 Department of Trauma Surgery, Amsterdam UMC, Meibergdreef 9, 1105 AZ Amsterdam, The Netherlands; 2 Amsterdam Movement Sciences, Musculoskeletal Health, De Boelelaan 1117, 1081 HV Amsterdam, The Netherlands; 3 Department of Epidemiology and Data Science, Amsterdam UMC, Meibergdreef 9, 1105 AZ Amsterdam, The Netherlands; 4 Amsterdam Public Health Research Institute, Methodology, Meibergdreef 9, 1105 AZ Amsterdam, The Netherlands

**Keywords:** Thresholds, Upper extremity injury, Vignette study

## Abstract

**Purpose:**

In this study we focused on gaining better interpretation of the PROMIS-Upper-Extremity v2.0 (PROMIS-UE v2.0) T-score scale for measuring upper extremity function in patients with upper extremity injuries (UEIs). This is fundamental in order to clearly understand the clinical relevance of these outcomes. The aim of this study was to identify interpretive thresholds for mild, moderate and severe upper extremity function problems for PROMIS-UE v2.0 T-scores, in patients with UEIs, by performing a vignettes study.

**Methods:**

A qualitative vignette study, based on the PROMIS-UE v2.0 and its item response theory model. Designed vignettes were tested in pilot expert- and patient focus groups. The vignettes were ranked by participants by functioning level and interpretive threshold values were proposed.

**Results:**

This resulted in consensus on the following interpretive thresholds: no problems—mild problems at T-score 45, mild problems—moderate problems at T-score 32.5 and moderate problems—severe problems at T-score 25. The thresholds provide additional information on interpretation of a larger range of the T-score scale, not only the evidently low or high T-scores.

**Conclusion:**

This study provides clinically meaningful interpretive thresholds for interpreting PROMIS-UE v2.0 T-scores in patients with UEI, supporting more patient-centred interpretation of treatment outcomes. We recommend using these interpretive thresholds in clinical practice to support the interpretation of patient-reported treatment outcomes and facilitate shared decision-making. Future research with regards to influencing factors on ranking of the vignettes should follow.

**Supplementary Information:**

The online version contains supplementary material available at https://doi.org/10.1007/s11136-026-04367-w.

## Abstract (plain language summary)

Upper limb injury is a common major problem for patients and society. Good and fast recovery is important. Recovery can be measured by questionnaires. Previous research showed that questionnaires can be useful in follow-up after injury, to involve the patient in their rehabilitation process and to measure the effect of treatment from the patient’s perspective. This study is needed for better understanding of the scores of a specific upper limb function questionnaire (PROMIS-Upper-Extremity (UE) v2.0). An important step in assessing the clinical relevance of the questionnaire results is to improve the interpretation of the scoring scale of this specific questionnaire (PROMIS-UE v2.0). This study designed hypothetical patients (vignettes) with different levels of function, based on hypothetical answers to selected questions of the PROMIS-UE v2.0. The main goal of this study was to identify thresholds based on the severity of functional problems (no—mild problems, mild—moderate problems, and moderate—severe problems). In a meeting with five patients and six clinicians, the vignettes were ordered from normal (high) to severely impaired (low) upper extremity function and cut-off values for mild, moderate, and severe scores were proposed. This study is a step towards better understanding of the PROMIS-UE v2.0 questionnaire, which will contribute to more patient-centred evaluation of treatment outcomes.

**Supplementary Information:**

The online version contains supplementary material available at https://doi.org/10.1007/s11136-026-04367-w.

## Purpose

Upper extremity injuries (UEIs) are a major problem for patients and society as it negatively affects work participation, health-related quality of life, mental health, and increases expenses for health care [1–3]. UEIs substantially impair daily functioning and quality of life, highlighting the importance of efficient and effective rehabilitation. Rehabilitation involves physical and psychosocial factors, a movement towards shared decision-making and patient-tailored care [4]. Rehabilitation can be assessed using Clinician-Reported Outcome Measures (ClinROMs) and Patient-Reported Outcome Measures (PROMs). ClinROMs such as grip strength, range of motion and X-rays, have been the gold standard for years. With growing emphasis on health-related quality of life and patient-tailored care, PROMs have gained prominence as tools to capture patients’ perceptions of function and well-being [4–6]. Rather than replacing ClinROMs, PROMs should complement them to enhance outcome assessment.

Commonly used PROMs for UEIs include the (Quick-)Disability of the Arm, Shoulder and Hand ((Q-)DASH) questionnaire [7, 8], the Patient-Rated Wrist Evaluation (PRWE) [9], the Michigan Hand Questionnaire (MHQ) [10], and the Patient-Reported Outcomes Measurement Information System Upper Extremity version 2.0 (PROMIS-UE v2.0) [11]. While PROMs can be time-consuming, PROMIS-UE v2.0 offers an efficient alternative through its short form and Computerized Adaptive Test (CAT) format.

### PROMIS-UE v2.0

The PROMIS-UE v2.0 is a 46-item bank assessing upper extremity (UE) function. Two 5-point response scales are used: “Unable to do” to “without any difficulty”; and “Cannot do” to “not at all”. The PROMIS-UE v2.0 is based on Item Response Theory (IRT) and uses a T-score metric, where a score of 50 represents the mean and 10 the standard deviation of a reference population. T-scores below 50 indicate worse, and scores above 50 better, upper extremity function relative to the reference population. As the PROMIS-UE v2.0 is based on IRT, it can be used as CAT or short form. CAT uses an algorithm that selects the most informative items from the item bank, based on the individual’s response to previously administered items, until the predetermined precision is reached (± 4–7 items) [12, 13]. In this way, high measurement accuracy can be obtained with lower burden for the patient. The PROMIS-UE v2.0 item bank was translated into Dutch-Flemish language and validated in a Dutch sample of patients with UEI [13].

## Thresholds in UE function

A critical step toward assessing the clinical relevance of its outcomes, is to improve interpretation of the PROMIS-UE v2.0 T-score scale. One way to clarify this T-score scale and its clinical relevance for patients with UEI, is by developing interpretive severity thresholds for mild, moderate and severe problems in UE function. Such thresholds can be derived using a vignette-based ranking method. Vignettes are hypothetical cases loaded onto a designated T-score [14]. The aim of this study was to propose interpretive severity thresholds for mild, moderate and severe UE function for PROMIS-UE v2.0 T-scores, in patients with UEIs, by performing a vignettes study.

## Methods

To develop interpretive severity thresholds for PROMIS-UE v2.0 T-scores in patients with UEIs, the study was conducted at the Amsterdam UMC location VUmc, the Netherlands. Dispensation for medical ethical approval was granted by the local Medical Ethics Committee [2020.02]. Official PROMIS-UE v2.0 item names were used, and the full item bank can be requested at HealthMeasures.

This vignette study follows the six required steps: (1) predicting item responses, (2) identifying target locations, (3) designing vignettes, (4) pilot testing in expert focus group, (5) patient focus group, and (6) identifying thresholds [14].

## Step 1. Predicting item responses

Based on the IRT model, for each T-score the most likely response to each item of the PROMIS-UE v2.0 can be predicted. For all 46 items, the predicted item responses per T-score were obtained with permission from the Health Measures website (https://www.assessmentcenter.net/ac_scoringservice). Data were imported as CSV files into RStudio^®^ (version 4.2.1), where predicted item responses were computed using a custom R script [15]. A section of the table with predicted item responses and corresponding colour coding is shown in Supplement 1.

## Step 2. Identifying target locations

Eight target locations were identified on the T-score scale, from 15 to 50, for which vignettes were developed. Target locations between T-scores of 15 and 50 were selected because the study aimed to identify clinically meaningful differences in UE function in the clinical range. A PROMIS-UE v2.0 T-score of 50 corresponds to the general population mean. Examination of predicted item responses demonstrated floor and ceiling effects at the extremes of the PROMIS-UE v2.0 scale. At T-scores ≥50, all items were associated with the highest response category, whereas at T-scores ≤15, all items were associated with the lowest response category. Consequently, vignettes outside this range would provide limited discrimination between severity levels. Collectively, these considerations informed the decision to select a T-score range of 15 to 50 for vignette development. Each vignette consisted of five items from the item bank [14]. For each selected T-score, a combination of five items and their responses (1. ‘Unable to do’ – 5. ‘Without any difficulty’) were used to write a vignette. For example, vignette A represented a hypothetical patient with a T-score of 15. For all five selected PROMIS-UE v2.0 items, this hypothetical patient scored *(1) ‘unable to do’*. Every subsequent vignette represented a T-score that was 0.5 SD higher than the one before. For example, subsequent vignette B represented a hypothetical patient with a T-score of 20. For this hypothetical patient it was possible to perform three out of five items with *(2) much difficulty* and for two out of five items the patients scored *(1) unable to do*.

### Step 3. Designing vignettes

Given that different UE activities and functional limitations may have varying impacts on health-related quality of life, we aimed to develop balanced and comparable vignettes. To achieve this, the 46 PROMIS-UE v2.0 items were categorized into four domains: (1) *personal hygiene*,* (2) ability to dress oneself*,* (3) ability to perform household tasks*,* and (4) strength/heavy lifting related*. In order to represent all domains of UE function, each vignette was designed to contain items from all categories. To maximize variability, three different vignettes per T-score were constructed which resulted in three series of eight vignettes. To enhance participant recognition of the vignettes, all three vignette sets were developed in a male, female and non-binary gender versions. Initially for the expert pilot focus group, the vignettes were named in alphabetical order based on descending T-score (names starting with A – H). However, for the patient focus group the names were assigned randomly to prevent selection bias. All vignettes were printed on cards so participants could better visualize the situations during the focus groups.

## Step 4. Expert pilot focus group

Focus groups generally include 6–12 participants [16]. For the expert focus group, we decided to include six clinicians (two trauma surgeons, one surgical resident, two physical- and one hand therapist) that work with patients with UEIs. The clinicians worked at Amsterdam UMC location VUmc and Spaarne Gasthuis, they were contacted personally or by e-mail and participation was voluntary. The vignettes were pilot tested with experts to refine wording, ensure clarity of instructions and objectives, and for vignettes ranking. Based on their feedback, minor adjustments were made prior to use in the patient focus group (step 5).

## Step 5. Patient focus group

Twelve patients were invited, and five patients participated in the focus group (see Table 1 for patient characteristics). Inclusion criteria included age > 18 years, UEI within the past five years, sufficient Dutch language proficiency, and the ability to speak in a group setting. Patients with chronic conditions were excluded. To ensure sample diversity, patients were selected based on variation in gender, age, UEI, education, and physical demanding occupations. Furthermore, the five-year inclusion window was intended to capture a range of perspectives, including both patients who were still actively recovering and those reflecting on their recovery retrospectively, thereby incorporating diverse experiences of UEI into the threshold-setting process. Participants were explicitly instructed not to relate the vignettes to their own injury experiences, but instead to evaluate the severity of the functional limitations described in each vignette. Eligible patients were recruited at the trauma surgery outpatient clinic of the Amsterdam UMC (location VUmc) and enrolled after providing informed consent. Participants did not receive financial compensation.

**Table 1:** Patient focus group participant characteristics

	Details	Total
Gender	M	3
	F	2
Age (years)	Range 30–76	Mean 57 (SD 19.1)
UEI (N = 5)	Proximal ulna fracture	1
	Clavicle fracture	2
	Digit 2–4 fracture	1
	Olecranon fracture	1
Trauma mechanism (*N* = 5)	Polytrauma	2
	Monotrauma	3
Treatment	Surgical	3
	Conservative	2
	Secondary surgical	2

### Step 6. Identifying thresholds

The following structure was used for both the expert and patient focus groups. First, as a warm-up exercise, an illustrated and simplified example was shown to the participants on how to rank “*cake recipes*” on a scale from “*easy to make”* to *“difficult to make”*, to facilitate understanding of ranking and threshold placement between vignettes [14]. Second, participants individually ranked the vignettes from normal (high) to severely impaired (low) upper extremity function. Third, the individual rankings were discussed within the groups to reach consensus. Fourth, participants individually placed thresholds between vignettes representing normal vs. mild, mild vs. moderate, and moderate vs. severe upper extremity function problems. At last, another group discussion followed to reach consensus on the thresholds. Set 3 would only be presented to the patients if no consensus was reached with set 1 and 2. The moderators closely guided the focus group sessions. During the individual ranking and threshold placement exercises, participants completed their assessments independently and discussion was not permitted, ensuring that initial judgments were uninfluenced by other participants. During the subsequent group discussions, moderators actively facilitated balanced participation and encouraged all participants to contribute equally. Participants were invited to consider alternative viewpoints while maintaining their own independent judgments, thereby minimizing the potential influence of dominant individuals and supporting the development of robust consensus-based threshold recommendations. The focus group took approximately 90 min. A focus group protocol can be requested. The focus groups were audio-recorded and field notes were made by the two moderators. All points of feedback were transcribed and evaluated.

## Results

### Development vignettes

**Fig. 1:**
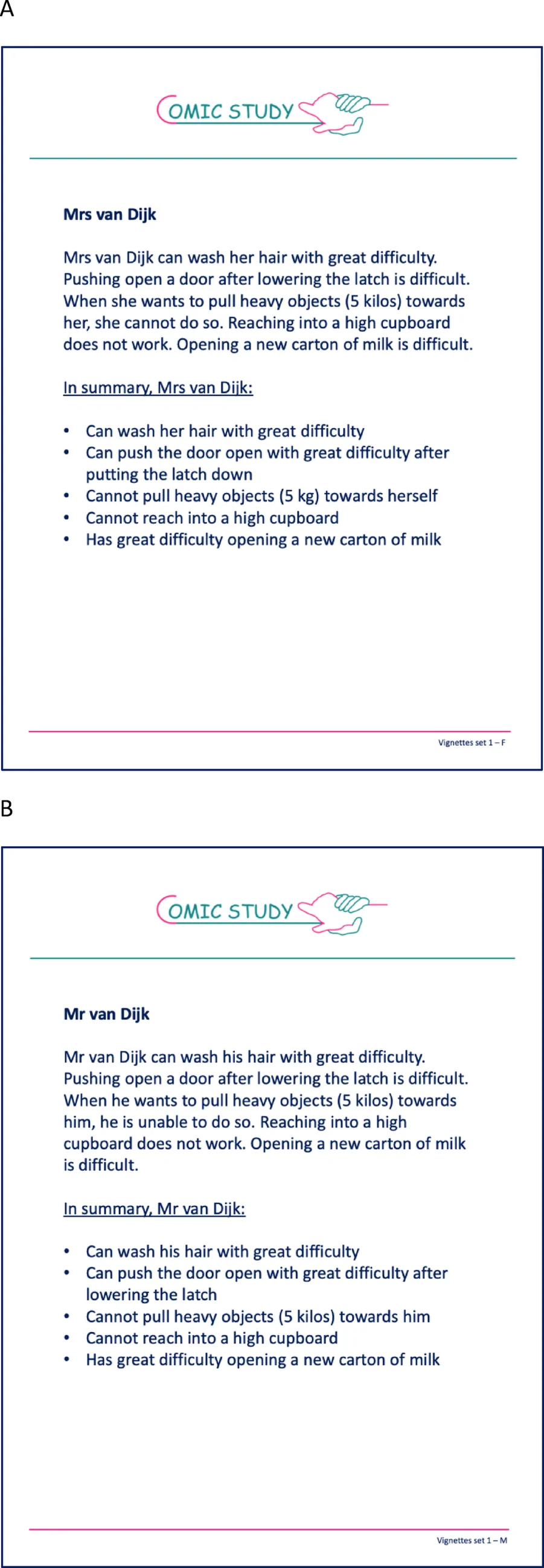
**A** Example of the female vignettes issued (translated to the English language). **B** Example of the male vignettes issued (translated to the English language)

Three sets (1–3) of eight vignettes (A-H) were developed. Supplement 2 shows an overview of the prototype vignettes with their item response scores per target location. All sets used identical item score combinations, except vignette 1C (T-score 25) and vignette 1D/2D/3D (T-score 30). Vignette 1C scored (1,2,2,2,3) versus (1,2,2,3,3) in 2C/3C. Vignette 1D scored 2,2,3,3,4, 2D 2,2,3,3,3 and 3D 2,2,2,3,3. Figure 1A/B shows two translated examples of the Dutch vignettes.

### Expert focus group

For all three sets consensus was reached. The expert ranking was in accordance with the predetermined T-score scale (Table 2). Threshold for normal—mild impairment was placed between T-scores 45–50 across all sets. For mild—moderate, thresholds ranged from 30 to 40 (Set 1: 35–40, Sets 2/3: 30–35). Moderate—severe impairment thresholds fell between 20 and 30, with Set 1/3 at 25–30 and set 2 at 20–25 (Table 2).

**Table 2:**
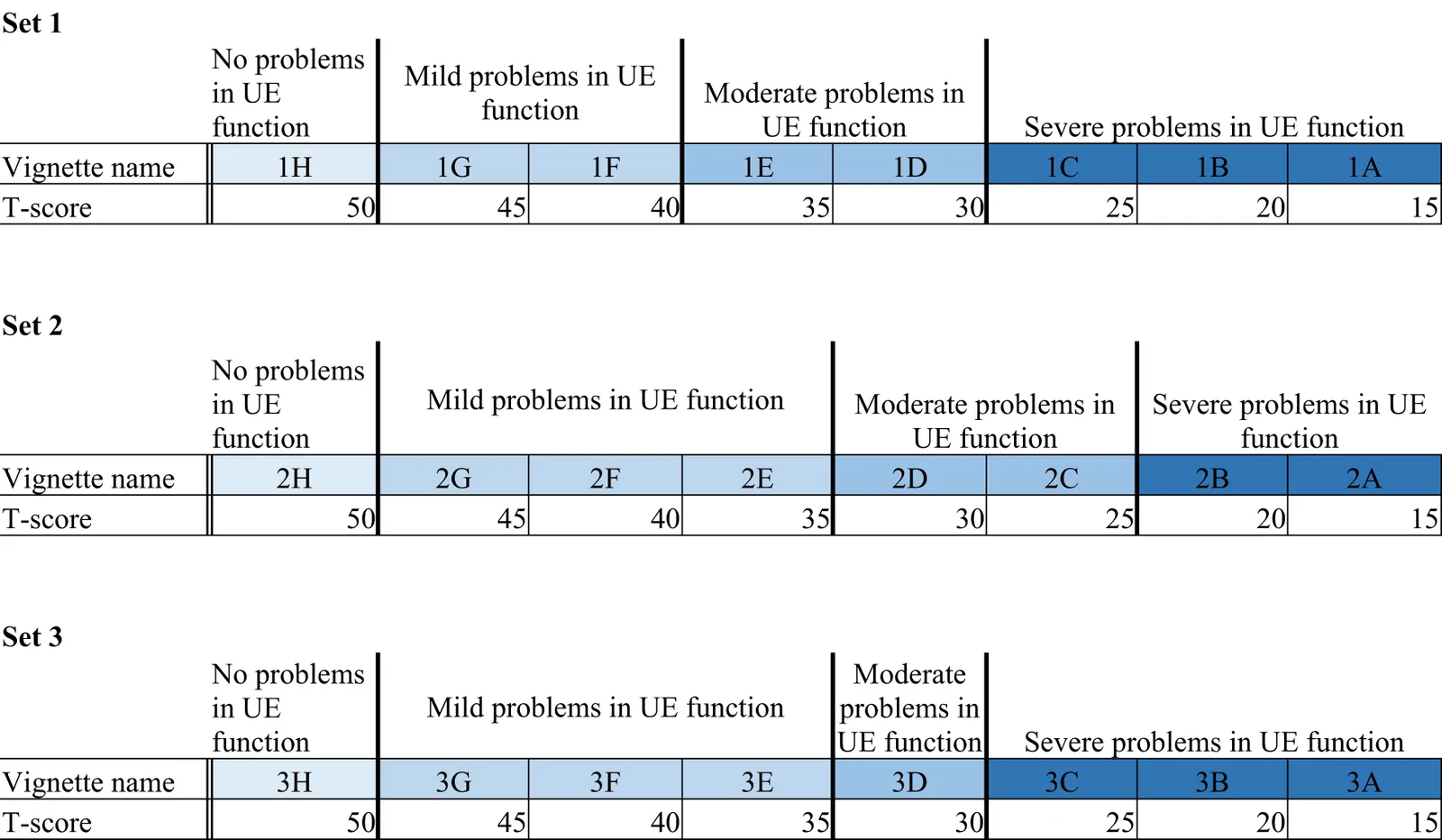
Expert panel consensus about ranking and thresholds for vignette set 1, 2 and 3 with corresponding names and T-scores

### Modifications vignettes

The vignettes were revised based on feedback from the expert focus group before presentation to patients. First, item PFM18 about swinging a tennis racket for five minutes was replaced due to unclear and non-identifiable wording. For Sets 1/3, in vignettes 1E/3E (T-score 35) it was substituted with PFA17 about *reaching into a high cupboard* and in vignettes 1F/3F (T-score 40) with PFA29r1 about *ability to pull 5 kg*. Second, as the item response was different for vignette 1C (T-score 25) compared to the other T-score 25 vignettes (2C/3C), it was switched from 2,3,2,2,1 to 2,3,2,3,1. Last, some clinicians found the vignettes phrasing too staccato, while others preferred the clarity of short sentences. For the patient focus group, vignettes were rewritten using official PROMIS-UE v2.0 translations. To minimize selection bias, vignette labels were randomized.

### Patient focus group

Consensus on ranking and thresholds was achieved for Sets 1 and 2, aligning with the T-score scale. As consensus was achieved easily, Set 3 was not necessary. The final advised interpretive thresholds were derived from the consensus threshold placements across the two patient vignette sets. Within each vignette set, threshold values were calculated as the midpoint between the two adjacent vignette T-scores defining each severity threshold. The final advised thresholds represent the average of the corresponding threshold values across vignette sets 1 and 2. For example, for the mild–moderate threshold, the midpoint between T-scores 30 and 35 was 32.5 in both vignette sets, resulting in a final advised threshold of T = 32.5. Thresholds were placed at T-score 45 (no—mild problems), 32.5 (mild—moderate problems), and 25 (moderate—severe problems), as detailed in Tables 3 and 4.

**Table 3:**
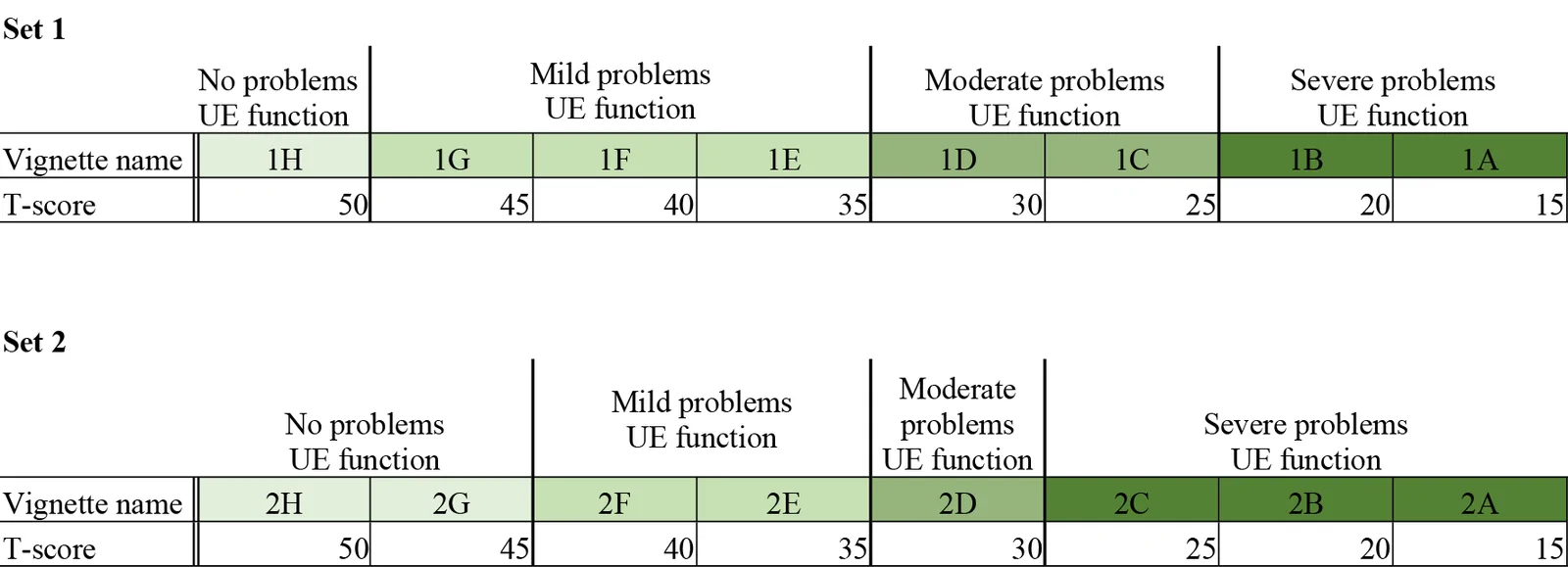
Patient focus group ranking and thresholds for vignettes set 1 and 2 with corresponding names, numbers, T-scores and thresholds between groups

**Table 4:** Patient focus group, advised cut-offs

	T-score
No problems–mild problems	45
Mild problems–moderate problems	32.5
Moderate problems–severe problems	25

### Qualitative data focus group

In addition to rankings and thresholds, the focus group yielded valuable qualitative data. Consensus regarding both vignette ranking and severity threshold placement was achieved, with no substantial disagreements observed during the focus group discussions. Consensus on the lowest and highest vignettes (1A/1H and 2A/2H) was reached quickly. Except for minor variations in adjacent vignette orderings, rankings were consistent. With minimal moderator guidance, full consensus was easily achieved. Threshold consensus required slightly more discussion.

Regarding the normal – mild threshold, participants discussed different perceptions of what constitutes ‘healthy’. Certain activities, such as drying one’s back (PFA34) and passing 10 kg of food (PFM16), were challenging for some patients even in good health, highlighting individual variation in functional norms. Besides, patients varied in valuing strength versus fine motor skills, with polytrauma patients favouring the latter due to its relevance for independence in daily activities such as showering and eating. For the moderate – severe threshold, two key factors emerged: social support and the value of independence. Patients without support perceived greater burden, and those valuing independence rated impairments as more severe. At last, participants indicated that additional contextual details such as age and gender, were needed to rank vignettes accurately. No substantial disagreements were observed during the focus group discussions. Participants actively shared and explained their perspectives while remaining open for alternative viewpoints. The discussions were constructive in nature, and consensus regarding vignette ranking and threshold placement was reached without difficulty.

While patients and experts showed a high degree of agreement in their vignette rankings, some differences in threshold placement were observed. For Set 1, patients and experts selected the same threshold for the transition from normal to mild problems (T = 45–50). For the remaining thresholds, experts consistently selected higher T-scores, corresponding to better upper extremity function, than patients. These differences were limited to a single vignette (5 T-score points). For Set 2, patients and experts selected the same threshold for the transition from mild to moderate problems (T = 30–35). The remaining thresholds differed between groups, but again by no more than one vignette (5 T-score points).

## Discussion

This study aimed to propose interpretive severity thresholds for mild, moderate and severe UE function problems for the PROMIS-UE v2.0 in patients with UEIs, by performing a vignettes study. The proposed interpretive thresholds provide additional information on interpretation of a larger range of the T-score scale supporting clinical interpretation, not only the evidently low or high T-scores. This resulted in consensus on the following interpretive T-score thresholds: no—mild problems at 45, mild—moderate problems at 32.5 and moderate—severe problems at 25.

### Vignette ranking

Consensus regarding ranking was reached easily at both expert- and patient focus groups. The alignment between vignette rankings and T-scores required no discussion, suggesting either that the vignettes were highly relatable (due to realistic design elements such as gender specific tasks) or that functional differences were too obvious. The extremes were easy to compare, in contrast to the vignettes more in the middle. Based on group interaction, we do not assume that the functional differences between the vignettes were too obvious. Besides, some items were used in more vignettes within the same set, what might have facilitated ranking as well. In this case, participants most likely compared the item responses to each other, rather than judging the clinical content of the vignette [14]. Although the variety of eligible items was limited during vignette development, the repetition of items was prevented as much as possible.

### Vignette thresholds

Both expert- and patient focus groups showed small differences in interpretive thresholds placement between sets, despite identical T-score classifications. In the expert focus group, in Sets 1 and 3 the threshold between moderate and severe problems was placed between T-scores 25–30, while for Set 2 it was between 20 and 25. The threshold between mild and moderate problems was placed between T-scores 35–40 for Set 1, and between T-scores 30–35 for Sets 2 and 3. In the patient focus group differences were observed as well, the normal – mild problems threshold was placed at T-score 45–50 in Set 1, and 40–45 in Set 2. The threshold between moderate and severe problems was placed at T-score 20–25 for Set 1 and 25–30 for Set 2. As consensus was reached, no thresholds were placed for Set 3.

Although patients and experts largely agreed on vignette rankings, some differences were observed in interpretive threshold placement. This may reflect differences in how patients and clinicians perceive upper extremity function and define severity levels. Furthermore, the replacement of item PFM18 may have influenced interpretive threshold placement, as the original item was considered difficult to interpret by patients and represented a relatively high-performance activity according to experts.

As the vignette T-scores (the vignette difficulty based on item score combinations) were comparable among sets, the explanation could lay within their clinical content. We hypothesize that these differences can be explained by Differential Item Functioning (DIF). DIF occurs when the probability of giving a certain response to an item is different across people from different groups. Recent research reported DIF by UEI location, with hand/wrist injuries linked to greater difficulty in fine tactile tasks despite similar overall UE function [6]. The same applies to patients with arm/shoulder injury, what seems linked to greater difficulty in strength related tasks despite similar overall level of UE function [6]. In the focus groups, these differences emerged as a point of discussion around different value placement on fine and gross motor skills. For example, polytrauma patients favoured fine motor skills due to its relevance for independence in daily activities such as showering and eating. These findings indicate that patient characteristics, especially individual experiences and values (i.e., their frame of reference), influence perceived UE function, functional limitations, and recovery. In addition to potential DIF across patient subgroups, perceptions of severity may be influenced by contextual factors such as social support, independence, and personal priorities. As these factors are not captured by PROMIS-UE T-scores, the proposed thresholds should be considered interpretive anchors that support, rather than replace, individualized clinical judgment. It is therefore important that future vignettes studies aim to minimize DIF, for example by ensuring a balanced distribution of fine tactile and strength items across vignettes and sets, and take the patients’ perspective and perceptions into account as well. This ensures equal relevance of the vignettes for both hand/wrist and arm/shoulder injury patients.

### In perspective

As vignettes in the present study were constructed at whole-number T-score locations, the resulting thresholds were expressed as values ending in .5. Future studies may consider using intermediate T-score locations, similar to the approach adopted by Rothrock et al., to facilitate whole-number threshold recommendations [17]. In the present study, vignettes were constructed at 5-point T-score intervals, which limited threshold placement to increments of five points. Future studies may consider developing vignettes at smaller T-score intervals to allow for more precise threshold recommendations. For example, Rothrock et al. addressed areas of uncertainty in threshold placement by developing and evaluating additional vignettes with intermediate T-scores between previously tested locations[17; 18]. A similar approach could be considered in future research to further refine PROMIS-UE severity thresholds in patients with UEI. A notable finding was the rapid ceiling effect observed when generating the predicted item responses. Maximum item scores were already reached at approximately T = 50, limiting the ability to construct distinguishable vignettes at higher levels of upper extremity function. Rothrock et al. (2023) reported a similar observation and suggested that this characteristic may make PROMIS-UE scores less directly comparable to those of other PROMIS item banks [17]. The severity thresholds identified in the present study were broadly consistent with those reported by Rothrock et al. (2023) (T = 40, 30, 25/20), although some thresholds were slightly higher. One possible explanation is that the study population differed, as only two of the eleven patients included by Rothrock et al. (2023) had upper extremity injuries [17]. Rothrock et al. (2025) showed comparable results with T = 40, 30 and 23 (and unanimity was not forced) as well, while they only included clinicians or patients with lower extremity injuries [18]. In contrast, our study exclusively included patients with upper extremity injuries, which may have influenced perceptions of functional severity and threshold placement.

### Strengths and limitations

The focus groups followed a clear protocol, suitable participants were invited, and rankings and interpretive threshold placement were decided carefully. The patient focus group was diverse in several aspects (age, gender, educational level and type of UEI), providing a broad range of patient perspectives, except with respect to treatment (conservative vs. surgical). The size of the focus group (*N* = 5) was considered both a strength and limitation. With a smaller group, the incentive to express your thoughts is greater, especially for people who normally avoid being in the spotlight. It is also easier for the researchers to accommodate everyone. However, a small group can also give less reliable results.

Our study has some points of discussion and limitations too. The PROMIS-UE v2.0 item bank allowed flexible item selection for vignette development. However, items with collapsed response options in the original calibration (PFA14r1, PFA16r1, PFA43r1, PFB15r1, PFB16r1, PFB19r1, PFB20r1, PFB21r1, PFB23r1, PFB31r1, PFB34, PFB37r1 and PFC8) were less suitable, as two different item response scores were possible for the same T-score. Excluding these items (over one third of the item bank) reduced flexibility and item variety for vignette development. Although the goal was to create identical vignette sets, vignette D varied slightly among sets. This did not affect interpretive threshold placement, highlighting the importance of clinical content over exact score matching. Ultimately, all patients underwent surgical treatment, either as primary treatment or following failure of initial conservative management, which may have influenced their perception of injury severity and recovery. In the expert focus group, the vignettes were distributed in order of T-score and alphabet, which may have possibly led to selection bias. On the other hand, after the focus group the experts said that this actually triggered them to pay extra attention, while others had not noticed the order, what resulted in a useful discussion. For the patient focus group, all forms of ranking were removed, minimising any selection bias. Besides, the validity of vignette-based methods depends on how accurately health states are described, though they cannot fully capture the patient experience [19]. Therefore, when designing vignettes, it is important to ensure that they describe a clear and relatively staccato case, to minimise misinterpretation [20, 21]. Another point of discussion is that patients with UEI within the previous five years were included to ensure familiarity with UE functional limitations and to capture perspectives from different stages of recovery. Although participants were instructed to base their judgements on the vignette descriptions rather than their own experiences, some degree of retrospective anchoring may have influenced perceptions of severity and threshold placement. Furthermore, the robustness of threshold placement to group dynamics was not formally evaluated. Several design features were implemented to support independent judgment, including individual ranking exercises prior to discussion and active moderation to encourage balanced participation. Nevertheless, the influence of group interaction on final consensus decisions cannot be completely excluded. As also suggested by Rothrock et al., it is possible that if unanimity in threshold placement were not pursued during the consensus process, greater variation in interpretive threshold recommendations among participants would remain [18]. This could indicate that some interpretive thresholds are more strongly supported by the study population than others [18]. This is an interesting methodological consideration and may warrant further investigation in future bookmarking studies. Furthermore, the impact of measurement uncertainty on threshold placement was not formally assessed. As PROMIS-UE scores are subject to measurement error, the proposed thresholds should be interpreted as interpretive severity thresholds rather than exact score boundaries.

#### Future perspectives

We recommend using the PROMIS-UE v2.0 interpretive severity thresholds to contextualize patient reported outcomes. Further data on T-scores and functional thresholds may provide clinical care and support transparency in national quality registries. In future studies, one should be aware that patient characteristics and individual values influence perceived UE function, limitations and recovery, what might influence ranking and threshold placement of vignettes as well. Testing vignettes in larger samples can be suggested, but more different focus groups with patients with matching characteristics could also provide more information on thresholds.

## Supplementary Information

Below is the link to the electronic supplementary material.

Supplementary Material 1

## Data Availability

All relevant data generated or analysed during this study is included in this article and its figures and tables.

## References

[1] Bernstein, D. N., Gruber, J. S., Merchan, N., Garcia, J., Harper, C. M., & Rozental, T. D. (2021). What factors are associated with increased financial burden and high financial worry for patients undergoing common hand procedures? *Clinical Orthopaedics And Related Research*, *479*(6), 1227–1234. 10.1097/CORR.0000000000001616 PMC8133202 33394757

[2] Polinder, S., Haagsma, J., Panneman, M., Scholten, A., Brugmans, M., & Van Beeck, E. (2016). The economic burden of injury: Health care and productivity costs of injuries in the Netherlands. *Accident Analysis And Prevention*, *93*, 92–100. 10.1016/j.aap.2016.04.003 27177394

[3] O’Hara, N. N., Isaac, M., Slobogean, G. P., & Klazinga, N. S. (2020). The socioeconomic impact of orthopaedic trauma: A systematic review and meta-analysis. *PLoS One*, 15(1), e0227907. 10.1371/journal.pone.0227907 PMC6961943 31940334

[4] Weldring, T., & Smith, S. M. (2013). Patient-reported outcomes (PROs) and patient-reported outcome measures (PROMs). *Health Serv Insights*, *6*, 61–68. 10.4137/HSI.S11093 PMC4089835 25114561

[5] Beerekamp, M. S. H., de Muinck Keizer, R. J. O., Schep, N. W. L., Ubbink, D. T., Panneman, M. J. M., & Goslings, J. C. (2017). Epidemiology of extremity fractures in The Netherlands. *Injury*, *48*(7), 1355–1362. 10.1016/j.injury.2017.04.047 28487101

[6] Lameijer, C. M., van Bruggen, S. G. J., Haan, E. J. A., Van Deurzen, D. F. P., Van der Elst, K., Stouten, V., Kaat, A. J., Roorda, L. D., & Terwee, C. B. (2020). Graded response model fit, measurement invariance and (comparative) precision of the Dutch-Flemish PROMIS^®^ Upper Extremity V2.0 item bank in patients with upper extremity disorders. *Bmc Musculoskeletal Disorders*, *21*(1), 170. 10.1186/s12891-020-3178-8 PMC7077019 32178644

[7] Hudak, P. L., Amadio, P. C., Bombardier, C., Beaton, D., Cole, D., Davis, A., Hawker, G., Katz, J. N., Makela, M., Marx, R. G., Punnett, L., & Wright, J. (1996). Development of an upper extremity outcome measure: The DASH (Disabilities of the Arm, Shoulder, and Hand).* American Journal of Industrial Medicine 30*(3), 372–372. 10.1002/(SICI)1097-0274(199606)29:6<602::AID-AJIM4>3.0.CO;2-L 8773720

[8] Beaton, D. E., Wright, J. G., Katz, J. N., & Collaborative, U. E., G (2005). Development of the QuickDASH: Comparison of three item-reduction approaches. *Journal Of Bone And Joint Surgery. American Volume*, *87*(5), 1038–1046. 10.2106/JBJS.D.02060 15866967

[9] MacDermid, J. (1996). Development of a scale for patient rating of wrist pain and disability. *Journal of Hand Therapy*, *9*(2), 178–183. 10.1016/s0894-1130(96)80076-7 8784681

[10] Chung, K. C., Pillsbury, M. S., Walters, M. R., & Hayward, R. A. (1998). Reliability and validity testing of the Michigan hand outcomes questionnaire. *J Hand Surg Am*, *23*(4), 575–587. 10.1016/S0363-5023(98)80042-7 9708370

[11] Tyser, A. R., Beckmann, J., Franklin, J. D., Cheng, C., Hon, S. D., Wang, A., & Hung, M. (2014). Evaluation of the PROMIS physical function computer adaptive test in the upper extremity. *J Hand Surg Am*, *39*(10), 2047–2051. e2044. 10.1016/j.jhsa.2014.06.130 25135249

[12] Reeve, B. B., Hays, R. D., Bjorner, J. B., Cook, K. F., Crane, P. K., Teresi, J. A., Thissen, D., Revicki, D. A., Weiss, D. J., Hambleton, R. K., Liu, H., Gershon, R., Reise, S. P., Lai, J. S., Cella, D., & Group, P. C. (2007). Psychometric evaluation and calibration of health-related quality of life item banks: plans for the patient-reported outcomes measurement information system (PROMIS). *Medical Care*, *45*(5 Suppl 1), S22–31. 10.1097/01.mlr.0000250483.85507.04 17443115

[13] van Bruggen, S. G. J., Lameijer, C. M., & Terwee, C. B. (2019). *Structural validity and construct validity of the Dutch-Flemish PROMIS((R)) physical function-upper extremity version 2.0 item bank in Dutch patients with upper extremity injuries* (pp. 1–9). Disabil Rehabil. 10.1080/09638288.2019.1651908 31411908

[14] Cook, K. F., Cella, D., & Reeve, B. B. (2019). PRO-bookmarking to estimate clinical thresholds for patient-reported symptoms and function. *Medical Care*, *57*(Suppl 5 Suppl 1), S13–s17. 10.1097/MLR.0000000000001087 30985591

[15] Morgan, E. M., Mara, C. A., Huang, B., Barnett, K., Carle, A. C., Farrell, J. E., & Cook, K. F. (2017). Establishing clinical meaning and defining important differences for patient-reported outcomes measurement information system (PROMIS(^®^)) measures in juvenile idiopathic arthritis using standard setting with patients, parents, and providers. *Quality Of Life Research*, *26*(3), 565–586. 10.1007/s11136-016-1468-2 PMC5311023 27913986

[16] Moser, A., & Korstjens, I. (2018). Series: Practical guidance to qualitative research. Part 3: Sampling, data collection and analysis. *The European Journal Of General Practice*, *24*(1), 9–18. 10.1080/13814788.2017.1375091 PMC5774281 29199486

[17] Rothrock, N. E., Wilson, S. A., Heng, M., Hodor, A., Joeris, A., Kaat, A. J., McKelvey, K., Schalet, B. D., & Vrahas, M. (2023). Using bookmarking methods with orthopedic clinicians and patients with fractures produces score interpretation labels for patient-reported outcome measures. *Quality Of Life Research*, *32*(10), 2779–2787. 10.1007/s11136-023-03439-5 PMC10474193 37227662

[18] Rothrock, N. E., Drandarov, A., Kaat, A. J., Mosher, H., Prado, J., & Heng, M. (2025). Clarifying thresholds for defining levels of physical function and pain interference using bookmarking in orthopaedics. *Quality Of Life Research*, *34*(4), 1159–1166. 10.1007/s11136-024-03881-z PMC11982084 39718724

[19] Wright, A., Hannon, J., Hegedus, E. J., & Kavchak, A. E. (2012). Clinimetrics corner: a closer look at the minimal clinically important difference (MCID). *The Journal Of Manual & Manipulative Therapy*, *20*(3), 160–166. 10.1179/2042618612Y.0000000001 PMC3419574 23904756

[20] Matza, L. S., Stewart, K. D., Lloyd, A. J., Rowen, D., & Brazier, J. E. (2021). Vignette-based utilities: usefulness, limitations, and methodological recommendations. *Value In Health : The Journal Of The International Society For Pharmacoeconomics And Outcomes Research*, *24*(6), 812–821. 10.1016/j.jval.2020.12.017 34119079

[21] Sheringham, J., Kuhn, I., & Burt, J. (2021). The use of experimental vignette studies to identify drivers of variations in the delivery of health care: a scoping review. *Bmc Medical Research Methodology*, *21*(1), 81. 10.1186/s12874-021-01247-4 PMC8061048 33888077

