# Supplementary material for: Deciding on thresholds for clinical relevance of PROMIS-upper extremity v2.0 in patients with upper extremity injury
Source: Qual Life Res. 2026 Aug 29;35(10):275. doi: 10.1007/s11136-026-04367-w (PMC13526136; doi:10.1007/s11136-026-04367-w)
Supplement: Supplementary file 1 [file 11136_2026_4367_MOESM1_ESM.docx]

**Supplement 1.** Predicted item responses per T-score^a^

| **Item** | **10** | **15** | **20** | **25** | **30** | **35** | **40** | **45** | **50** |
| --- | --- | --- | --- | --- | --- | --- | --- | --- | --- |
| Able to reach into high cupboard | 1 | 1 | 1 | 2 | 3 | 4 | 4 | 5 | 5 |
| Able to pound nail with hammer | 1 | 1 | 1 | 2 | 3 | 4 | 5 | 5 | 5 |
| Able to cut food using eating utensils | 1 | 1 | 2 | 3 | 4 | 5 | 5 | 5 | 5 |
| Able to open can with hand can opener | 1 | 1 | 2 | 2 | 3 | 4 | 5 | 5 | 5 |
| Able to pull 5kg/10 pounds object toward yourself | 1 | 1 | 1 | 2 | 3 | 4 | 4 | 5 | 5 |
| Able to wash back | 1 | 1 | 1 | 1 | 2 | 3 | 4 | 5 | 5 |
| Able to open and close zipper | 1 | 1 | 2 | 3 | 4 | 5 | 5 | 5 | 5 |
| Able to put on and take off coat | 1 | 1 | 2 | 3 | 4 | 4 | 5 | 5 | 5 |
| Able to dry back with towel | 1 | 1 | 1 | 2 | 3 | 4 | 5 | 5 | 5 |
| Able to turn key in lock | 1 | 1 | 2 | 3 | 4 | 5 | 5 | 5 | 5 |

^a^ With scores being: 1 = unable to do, 2 = with much difficulty, 3 = with some difficulty, 4 = with a little difficulty, 5 = without any difficulty.

**Supplement 2**. Items per vignette

**Vignettes Set 1**:

| **Target locations** | **15** | **20** | **25** | **30** | **35** | **40** | **45** | **50** |
| --- | --- | --- | --- | --- | --- | --- | --- | --- |
| **Vignette name** | 1A | 1B | 1C | 1D | 1E | 1F | 1G | 1H |
| **Item responses** | 1,1,1,1,1 | 1,1,2,2,2 | 2,3,2,3,1 | 2,3,4,3,2 | 4,4,3,3,3 | 4,5,5,4,3 | 5,5,5,4,4 | 5,5,5,5,5 |
| **Items** | PFA40  PFA48  PFA54  PFB41  PFB56r1 | PFA17  PFA29r1  PFB25  PFB26  PFB30 | PFA28  PFA36  PFA38  PFB13  PFM2 | PFA34  PFB11  PFB27  PFB39r1  PFM16 | PFB22  PFB36  PFM2  PFM16  PFA17 | PFA17  PFA28  PFA50  PFB39r1  PFA29r1 | PFA20  PFA29r1  PFA44  PFM2  PFM16 | PFA18  PFB18  PFB36  PFB41  PFC43 |

**Vignettes Set 2**:

| **Target locations** | **15** | **20** | **25** | **30** | **35** | **40** | **45** | **50** |
| --- | --- | --- | --- | --- | --- | --- | --- | --- |
| **Vignette name** | 2A | 2B | 2C | 2D | 2E | 2F | 2G | 2H |
| **Item responses** | 1,1,1,1,1 | 2,2,1,2,1 | 2,1,3,3,2 | 3,3,3,2,2 | 4,4,3,3,3 | 4,4,5,5,3 | 5,5,5,4,4 | 5,5,5,5,5 |
| **Items** | PFA29r1  PFA36  PFB18  PFB22  PFC49 | PFA20  PFA50  PFB13  PFB36  PFB56r1 | PFA17  PFA34  PFA44  PFB26  PFC43 | PFA18  PFA28  PFA38  PFB28r1  PFM2 | PFA36  PFB30  PFM2  PFM16  PFA29r1 | PFA29r1  PFA34  PFA48  PFB36  PFM16 | PFA17  PFA35  PFA50  PFM2  PFM16 | PFA20  PFA40  PFA54  PFB26  PFB39r1 |

**Vignettes Set 3**:

| **Target locations** | **15** | **20** | **25** | **30** | **35** | **40** | **45** | **50** |
| --- | --- | --- | --- | --- | --- | --- | --- | --- |
| **Vignette name** | 3A | 3B | 3C | 3D | 3E | 3F | 3G | 3H |
| **Item responses** | 1,1,1,1,1 | 1,1,2,2,2 | 2,3,3,2,1 | 3,2,3,2,2 | 4,4,3,3,3 | 4,5,5,4,3 | 5,5,5,4,4 | 5,5,5,5,5 |
| **Items** | PFA18  PFA35  PFA38  PFB30  PFB39r1 | PFA17  PFA29r1  PFA36  PFB18  PFC43 | PFA28  PFA35  PFA50  PFB11  PFB28r1 | PFA17  PFA34  PFB13  PFM16  PFB28r1 | PFA28  PFA38  PFM2  PFM16  PFA17 | PFA34  PFA54  PFB30  PFB39r1  PFA29r1 | PFA29r1  PFA48  PFM2  PFM16  PFB27 | PFA20  PFA44  PFB25  PFB26  PFB56r1 |
